# Insight into the Nucleation Mechanism of *p*-Methoxybenzoic Acid in Ethanol-Water System from Metastable Zone Width

**DOI:** 10.3390/molecules27134085

**Published:** 2022-06-24

**Authors:** Guangle Wang, Zeren Shang, Mingdi Liu, Weibing Dong, Haichao Li, Haiqing Yin, Junbo Gong, Songgu Wu

**Affiliations:** 1 State Key Laboratory of Chemical Engineering, School of Chemical Engineering and Technology, Tianjin University, Tianjin 300072, China; guanglewang5018@tju.edu.cn (G.W.); shangzeren@tju.edu.cn (Z.S.); liumingdi@tju.edu.cn (M.L.); junbo_gong@tju.edu.cn (J.G.); 2 School of Chemistry and Chemical Engineering, Qinghai Minzu University, Xining 810007, China; wbdong@tju.edu.cn (W.D.); lihaichao@vip.163.com (H.L.); qhmdyhq@163.com (H.Y.)

**Keywords:** *p*-methoxybenzoic acid, MSZW, nucleation, nucleation kinetic parameter

## Abstract

The metastable zone width (MSZW) of *p*-methoxybenzoic acid (PMBA) in an ethanol-water system was measured using the polythermal method. The nucleation order *m* obtained by the Nývlt’s model indicates the nucleation of PMBA following a progressive nucleation mechanism at low saturation temperature (*m* = 3.18–7.50) and an instantaneous nucleation mechanism at high saturation temperature (*m* = 1.46–2.55). Then, combined with the metastable zone experiment and the Sangwal model, we found that the MSZW and the interfacial energy reached the maximum when the mass fraction of ethanol was 0.8, which resulted in the smallest crystal product size. Meanwhile, the maximum $r_{crit}$ and $\Delta G_{crit}$ obtained based on the modified Sangwal model indicating the PMBA needs to overcome a higher nucleation barrier in the ethanol mass fraction of 0.8. Finally, we proposed a preferential strategy for adjusting MSZW by correlating the interfacial energy with the change in ethanol mass fraction, saturation temperature, and cooling rate, respectively.

## 1. Introduction

As a common separation technology, solution crystallization is widely used in pharmaceutical, chemical, and material manufacturing [1,2,3]. In order to obtain products with purity and qualified physicochemical properties, it is necessary to regulate the crystallization process. As the initial stage of the crystallization process, nucleation has received extensive attention and is usually used as a significant and effective means to adjust product quality [4].

The metastable zone width as an optimal operating area for the crystallization process exerts great significance to the understanding of the nucleation mechanism and kinetics. The isothermal and polythermal methods are two common methods to determine nucleation kinetics [5]. The isothermal method is determined by measuring the induction period of nucleation, while the polythermal method is achieved by monitoring the nucleation temperature at a constant cooling rate. Lenka employed both the induction period method and the MSZW method to measure the nucleation kinetics of l-asparaginenohydrate [6]. The results showed that the nucleation parameters obtained by the induction period method and the MSZW method were consistent. Shiau used the integral method [7] to obtain the nucleation parameters in different systems based on classical nucleation theory using MSZW data, and the results were consistent with those obtained using the induction period method. Although the induction period method and the MSZW method have the same effect on the study of nucleation kinetics, only a limited number of studies have explored the nucleation behavior adopted the MSZW method.

In order to better control the crystallization process, numerous efforts have focused on the influencing factors of MSZW, such as the temperature path, saturation temperature, stirring intensity, solvent, and additive [8,9,10,11]. The solvent is indispensable in solution crystallization, and its type, composition, and properties may have an important impact on MSZW, so it is favored to explore the role of the solvent in the nucleation process. Yang revealed a competitive relationship between chemical driving force and saturation temperature in nucleation by measuring the MSZW of ethyl vanillin in three different solvents [12]. Xu studied the nucleation behavior of glutaric acid in different solvents with the metastable zone experiment and solution chemistry and revealed that the capacity of the hydrogen bond donor affects the width of the metastable zone [13]. Wang experimentally determined the MSZW of adipic acid in different polar solvents and confirmed the link between interfacial energy and the nucleation driving force [14]. Antisolvent crystallization is widely used in crystallization processes, and the effect of mixed solvents on solute nucleation has been reported, but most studies are based on the induction period method [15]. There are a few reports on the nucleation kinetics of mixed-solvent systems using the method of determining MSZW. In addition, the influence of saturation temperature, temperature path, and solution composition on MSZW needs further investigation.

It is worth noting that PMBA is a naturally occurring metabolite, also known as para-anisic acid or draconic acid. It manifests bioactivity, particularly as a tyrosinase inhibitor. It is also widely used as a drug in dermatological applications. Furthermore, it is a popular component of cosmetic and perfume compositions due to its capability to mask unpleasant smells [16]. The properties of the product after nucleation directly affect the solubility, transport, and bioavailability of the substance. Nonetheless, little literature has focused on nucleation behavior in the PMBA crystallization process.

The aim of this paper is to determine the MSZW of *p*-methoxybenzoic acid in an ethanol-water system by the polythermal method, and to further reveal the role of solvent composition on the nucleation of PMBA through different metastable zone models and classical nucleation theory. Finally, we try to provide a strategy for regulating the MSZW of PMBA by exploring the roles of saturation temperature, cooling rate, and solution composition in nucleation in detail.

## 2. Experiments and Methods

### 2.1. Materials

*p*-Methoxybenzoic acid (PMBA, 99%) and ethanol (≥99.5%) were purchased from Tianjin Kmart Chemical Technology Co. LTD (Tianjin, China). The deionized water used in the experiment was prepared using an ultra-pure water system in our laboratory.

### 2.2. Measurement of Solubility

The solubility of PMBA for different ethanol mass fractions in water was determined using the static method. Through preliminary experiments, we found that when the ethanol mass fraction in water was lower than 0.6, the solubility of PMBA was very small. This is not favorable for crystallization.

Therefore, we selected three solvent compositions with ethanol mass fractions in water (0.6, 0.8, and 1.0) in our experiment. Before the solubility measurement, solutions with ethanol mass fractions of 0.6, 0.8, and 1.0 were prepared and placed for use. Then, an excess amount of solid and about 50 mL mixed solvent with fixed ethanol/water mass ratio were added in a 100 mL glass container with a stopper. Afterwards, the glass container was sealed with sealing film and placed in a thermostatic shaker (type SHA-C, Jintan Tianjing Experimental Instrument Factory, Changzhou, China). Then, the thermostatic shaker was stirred continuously at a speed of 300 rpm for at least 24 h. Subsequently, stop the thermostatic shaker and keep the solution static for another 4 h to obtain a clear supernatant. After that, about 3 mL of supernatant was injected into a vial using a membrane syrin ge filter (0.22 μm). Finally, the vial was dried on a heating plate (333.15 K) after weighing. When the bottom is dry, move the vial to an oven at 333.15 K to dry until the two weighings (with an interval of more than 2 h) do not change. Five samples were collected under each condition, and the average of five measurements was taken as solubility data. The solubility (*x*) of PMBA in mixed solvents can be calculated as follows:

(1) $$x=\frac{m_{1}/152}{m_{1}/152+m_{2}/46+m_{3}/18}$$

where $m_{1}$, $m_{2}$ and $m_{3}$ represent the mass of PMBA, ethanol, and deionized water, respectively.

### 2.3. Metastable Zone Measurement Experiment for PMBA

**Figure 1:**
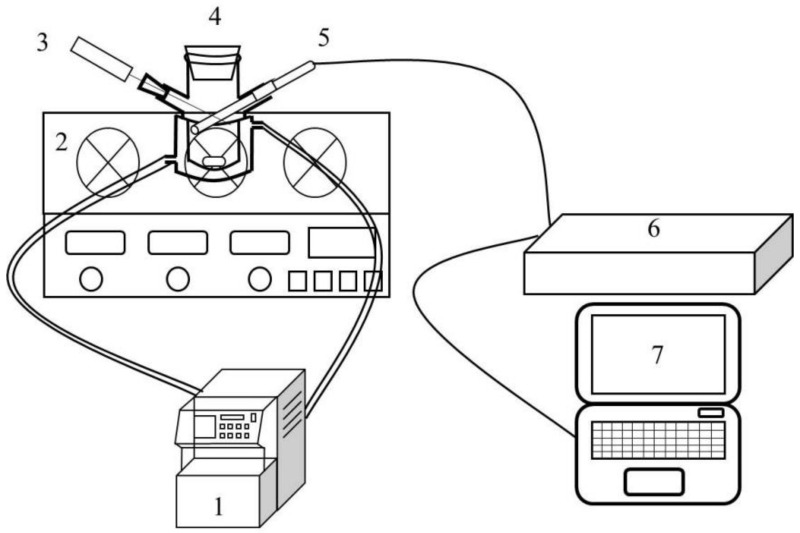
Metastable zone determination experimental flow chart (**1**: cooling circulating water machine, **2**: digital magnetic stirrer, **3**: digital thermometer, **4**: crystallizer, **5**: FBRM probe, **6**: FBRM workstation, **7**: computer).

**Figure 2:**
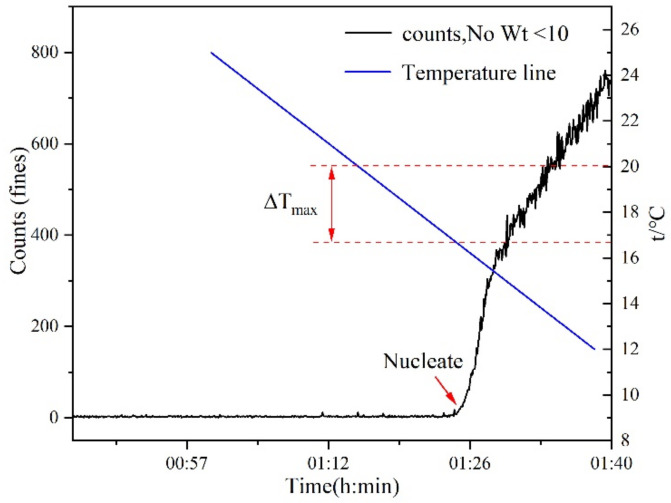
Schematic diagram of measuring the metastable zone by the Focused Beam Reflectance Meter (FBRM) (taking the saturation temperature of 293.15 K, stirring rate of 300 rpm, ethanol mass fraction of 0.6, and cooling rate of 20 K/h as an example).

The measurement of the metastable zone width was determined at three different saturation temperatures (293.15, 303.15, and 313.15 K). For each temperature, three different solvent mixtures (mass fraction of ethanol: 0.6, 0.8, and 1.0) were studied, while the cooling rate ranged from 10 K/h to 60 K/h. The jacketed crystallizer was filled with 50 g of solvent mixture and a given amount of PMBA, which depended on the solubility results. To allow the solute to dissolve completely, the solution was raised to a temperature above the saturation temperature of 5 K and maintained for about 1 h. The system was then cooled at a specified cooling rate and maintained at a stirring rate of 300 rpm. The spontaneous nucleation in the experiment was monitored by a focused beam reflectance measurement probe (FBRM), and the temperature was recorded using an electronic thermometer. Each experimental condition was repeated 3 times, and the results were averaged. The experiment flow chart of the measurement of metastable zone width is shown in Figure 1. In the measurement process, the recording frequency of FBRM was 5 s, and the chord length was selected from 1–1000 µm. When the particle number starts to rise, it is judged as the nucleation point, and the corresponding temperature is recorded. Figure 2 shows the diagram of FBRM monitoring nucleation at a saturation temperature of 293.15 K, stirring rate of 300 rpm, ethanol mass fraction of 0.6, and cooling rate of 20 K/h.

### 2.4. X-ray Powder Diffraction

The crystal forms of PMBA during metastable zone width measurements were evaluated using powder X-ray diffraction. All samples were measured with Cu Kα radiation (λ = 1.54046 Å) at 40 kV and 200 mA. The scan range was from 2° to 40°, and the scan rate was kept at 8° per min.

## 3. Theory

### 3.1. Nývlt’s Metastable Zone Model

Nývlt’s metastable zone model was proposed by Nývlt [17], which is a classical semi-empirical model to estimate nuclear power using the MSZW method. Due to its simple analysis, it has been widely used to correlate the relationship between cooling rate and MSZW [18]:

(2) $$\ln\Delta T_{max}=\frac{1-m}{m}\ln{\left(\frac{dc}{dT}\right)}_{T}+\frac{1}{m}\ln R-\frac{1}{m}\ln K$$

In the above equation, $\Delta T_{max}$ is the maximum temperature difference, m represents the nucleation order, ${\left(dc/dT\right)}_{T}$ stands for the temperature coefficient, R is the cooling rate, *K* is a coefficient, which is related to the studied system, nucleation and growth process and experimental method. It can be seen from the above equation that $\ln\Delta T_{max}$ has a linear relationship with lnR.

### 3.2. Sangwal Metastable Zone Model

In general, Equation (2) can fit the data of the metastable zone width well. However, as Nývlt’s method is semi-empirical, and the order and the nucleation rate constant have no physical meaning. Sangwal [19,20,21,22] developed a new method to analyze the MSZW determined by the polythermal method based on Nývlt’s method and classical 3D nucleation theory:

(3) $${\left(T_{0}/\Delta T_{max}\right)}^{2}=F_{1}\left(X+\ln T_{0}-\ln R\right)=F-F_{1}\ln R$$

Constant $F=F_{1}\left(X+\ln T_{0}\right)$, where,

(4) $$F_{1}=\left[\frac{3}{16\pi }{\left(\frac{k_{B}T_{1}}{\gamma \Omega^{2/3}}\right)}^{3}\times {\left(\frac{\Delta H_{S}}{R_{g}T_{1}}\right)}^{2}\right]$$

(5) $$X=\ln\left(\frac{A}{f}\frac{R_{g}T_{1}}{\Delta H_{S}}\right)$$

where $k_{B}$ stands for the Boltzmann constant, γ represents the solid-liquid interfacial energy, $\Delta H_{S}$ is the heat of dissolution, Ω is the volume of a molecule, $R_{g}$ is the gas constant, *A* is kinetic factor, *f* is the proportionality constant.

### 3.3. Modified Sangwal Metastable Zone Model

It is observed from Sangwal’s theory that the nucleation parameters are controlled by both $T_{0}$ and $T_{1}$. To further simplify Sangwal’s model, Equation (3) can be modified as follows [23]:

(6) $$\frac{{\left(\frac{T_{0}}{\Delta T_{max}}\right)}^{2}}{T_{0}-\Delta T_{max}}=M+N\ln\left[\frac{R}{T_{0}\left(T_{0}-\Delta T_{max}\right)}\right]$$

where the intercepts of *M* and slope *N* are denoted by:

(7) $$N=\left[\frac{-3}{16\pi }\frac{k_{B}{}^{3}}{\gamma^{3}\Omega^{2}}{\left(\frac{\Delta H_{S}}{R_{g}}\right)}^{2}\right]$$

(8) $$M=N\ln\left(\frac{f}{A}\frac{\Delta H_{S}}{R_{g}}\right)$$

As can be seen from the above equation, the parameters M and N of the simplified equation are independent of $T_{0}$ or $T_{1}$. The parameters A/f and γ can be easily obtained by regression of experimental data using Equation (6). Then the critical nuclei radius $r_{crit}$, the critical Gibbs free energy $\Delta G_{crit}$ and the nucleation rate J can be determined as follows:

(9) $$r_{crit}=\frac{2\gamma \Omega }{\Delta \mu }=\frac{2\gamma \Omega }{k_{B}T\ln S}$$

(10) $$\Delta G_{crit}=\frac{16\pi \gamma^{3}\Omega^{2}}{3{\left(k_{B}T\ln S\right)}^{2}}=\frac{16\pi \gamma^{3}\Omega^{2}}{3\Delta \mu^{2}}$$

(11) $$J=Aexp\left(-\frac{16\pi \gamma^{3}\Omega^{2}}{3k_{B}^{3}T_{1}^{3}{\left(\ln S\right)}^{2}}\right)$$

## 4. Results and Discussion

### 4.1. Solubility of PMBA in Ethanol Solution

**Figure 3:**
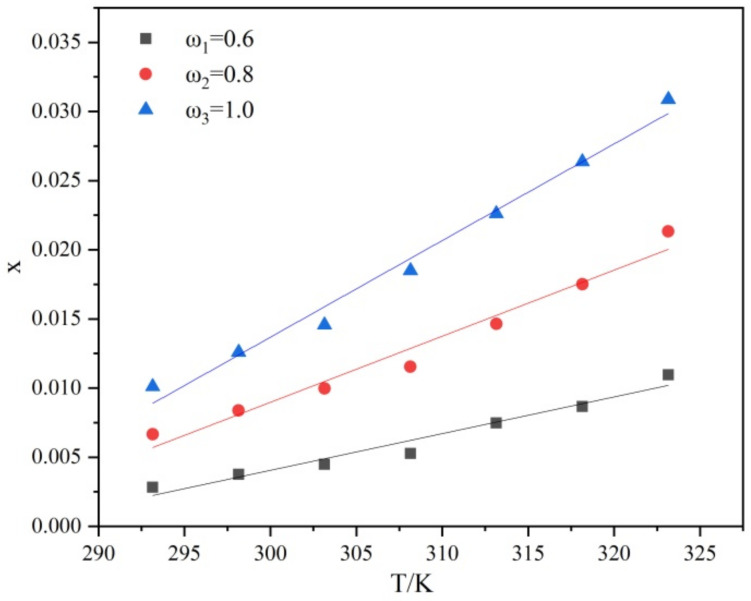
Relationship between solubility and temperature of PMBA under different ethanol mass fractions.

The solubility of PMBA under different ethanol mass fractions ω at 293.15 to 323.15 K is plotted in Figure 3.

**Figure 4:**
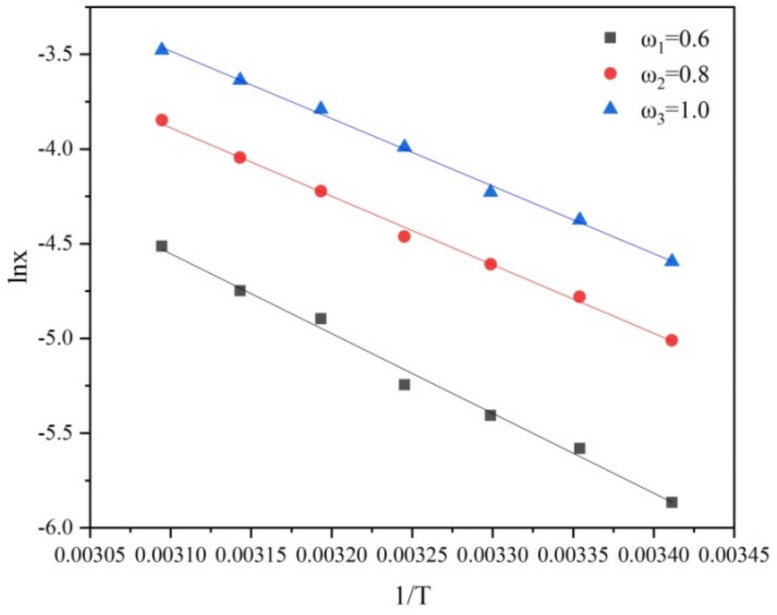
Solubility of PMBA at different ethanol mass fractions fitted by the Van’t Hoff equation.

As can be seen from Figure 3, the solubility of PMBA increased with the increase in temperature in the same ethanol composition. The solubility increased rapidly with the ethanol mass fraction. The correlation coefficient R^2^ of the fitted line is 0.9598, 0.9692, and 0.9853, respectively, indicating that the solubility has a good linear relationship with the temperature, and the temperature coefficient ${\left(dc/dT\right)}_{T}$ is a constant. Therefore, the Nývlt’s metastable zone model can be used to fit metastable zone data. At the same time, the solubility was fitted with the Van’t Hoff equation ($\ln x=-\frac{\Delta H_{s}}{R_{g}T}+\frac{\Delta S}{R_{g}}$) (as shown in Figure 4) to obtain the corresponding dissolution enthalpy $\Delta H_{s}$ and dissolution entropy ΔS. The results are shown in Table 1. $\Delta H_{s}$ can reflect the difference in the solvation intensity of solutes in different solution compositions to a certain extent. Table 1 shows that $\Delta H_{s}$ values in different ethanol mass fractions ranked as 0.6 > 0.8 > 1.0 in sequence. The results show that with an increase in the ethanol mass fraction, the enthalpy of dissolution decreases, and the solubility increases.

### 4.2. Solid-State Characterization of PMBA

**Figure 5:**
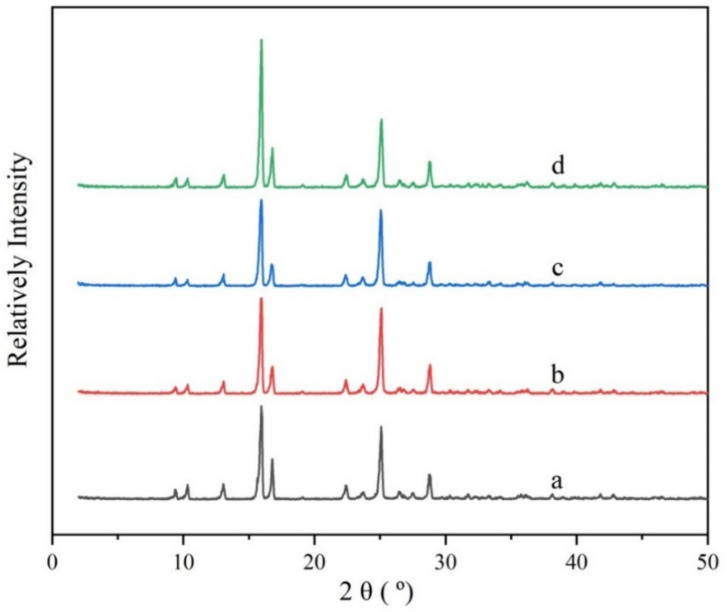
X-ray powder diffraction patterns of the PMBA solid samples. (**a**) is the raw material; (**b**–**d**) stands for the PXRD patterns of the samples obtained from solvent with ethanol mass fractions of 0.6, 0.8, and 1.0, respectively.

In order to verify whether polymorphs or solvates were generated during the experiment, the PMBA solids of raw materials and crystalline products in different mixed solvents were characterized by PXRD. The results are presented in Figure 5.

It can be seen that the crystal forms of typical solid products collected from the different composition solvents are the same as the raw materials.

### 4.3. Effects of Saturation Temperature, Cooling Rate, and Ethanol Mass Fractions on MSZW

The MSZW of PMBA in solvents of different ethanol mass fractions at different saturation temperatures and cooling rates are displayed in Table 2.

The results show that the MSZW vary with ω, $T_{0}$, and R. When the mass fraction of ethanol is the same, a low saturation temperature and fast cooling rate are beneficial for obtaining broad MSZW, which is consistent with other systems [24,25]. While the $T_{0}$ and R are constant; the MSZW widened and then narrowed with the increase in the ethanol mass fraction. To describe the changes of MSZW under the action of crystallization kinetics and thermodynamics, Nývlt’s model, Sangwl’s model, and the modified Sangwal model are selected for further discussion.

**Figure 6:**
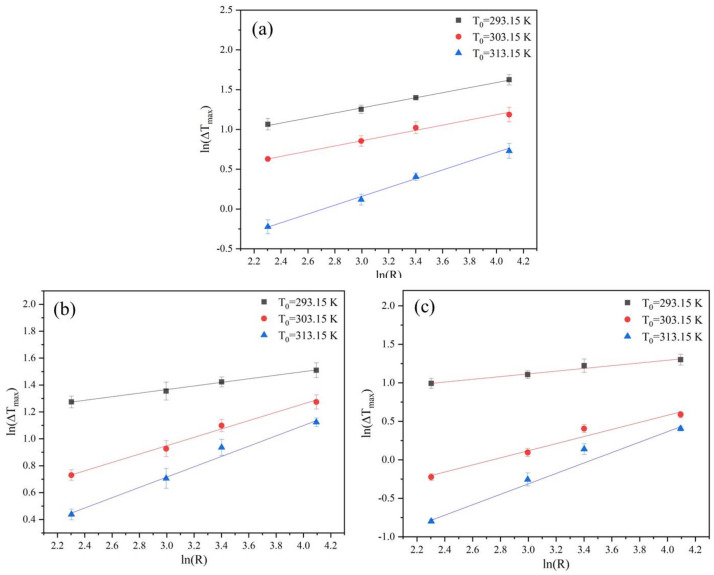
Relationship between MSZW and cooling rate at different saturation temperatures fitted by Nývlt’s model: (**a**) ethanol mass fraction of 0.6, (**b**) ethanol mass fraction of 0.8, and (**c**) ethanol mass fraction of 1.0.

The Nývlt’s model was employed to analyze the MSZW data, and the results are shown graphically in Figure 6. There is a linear relationship between $\ln\Delta T_{max}$ and lnR, indicating that MSZW data can be well described by Nývlt’s model. The *m* value (listed in Table 3) extracted from the Nývlt’s model is often used to estimate the nucleation mechanism roughly. The result suggests that *m* gradually decreases with an increase in the saturation temperature. At a saturation temperature of 293.15 K, the value of *m* is greater than 3, suggesting a progressive nucleation mechanism at a low saturation temperature [26]. At a saturation temperature of 313.15 K, the lower value of *m* between 1.46 and 2.55 indicates an instantaneous nucleation mechanism, which also indicates the existence of three-dimensional heterogeneous nucleation. When the saturation temperature is 303.15 K, the value of *m* is between 2.14 and 3.23. The value of *m* varies with the mass fraction of ethanol; when the mass fraction of ethanol is 1.0, the value of *m* is less than 3, and when the mass fraction of ethanol decreases, the *m* value is again greater than 3, which indicates that the nucleation mechanism is related to the mass fraction of ethanol.

**Figure 7:**
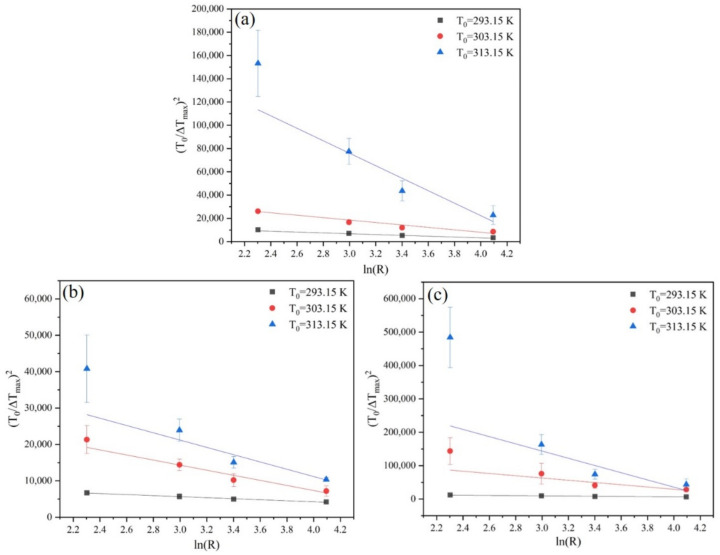
Relationship between MSZW and cooling rate at different saturation temperatures fitted by the Sangwal model: (**a**) ethanol mass fraction of 0.6, (**b**) ethanol mass fraction of 0.8, and (**c**) ethanol mass fraction of 1.0.

The cooling rate is the only variable parameter in the Nývlt’s model, while in the Sangwal model, both the cooling rate and the saturation temperature are variable parameters. The MSZW data fitted by the Sangwal model are shown in Figure 7. It can be seen from the fitting results that the Sangwal model can afford a good fit between ${\left(T_{0}/\Delta T_{max}\right)}^{2}$ and lnR. The greater slope of the fitting curve suggests that the influence of the cooling rate is greater at a higher saturation temperature. More importantly, the solid-liquid interfacial energy and nucleation kinetics factors can be easily obtained on the basis of fitting the MSZW data.

### 4.4. Nucleation Kinetic Parameters and Crystal Habit

The nucleation kinetic parameters were obtained by fitting the measured MSZW data based on the Sangwal model (Equation (7)). According to Equations (8) and (9), the interfacial energy γ and nucleation parameter *A*/*f* can be calculated, respectively, and the results are collected in Table 4 and Table 5.

As can be seen from Table 4 that the γ has a correlation with $T_{0}$ and R. As for the interfacial energy, it decreases significantly with the increase in saturation temperature, which corresponds to the value of MSZW, indicating that a high temperature is favorable for nucleation. Although γ decreases with the  R, the magnitude of the increase is small. The effect of the R on the γ value may be due to the decrease in the nucleation temperature due to the accelerated cooling rate. At a low saturation temperature, the nucleation process is dominated by primary nucleation. Secondary nucleation may dominate the crystallization process at a high saturation temperature due to the lower interfacial energy demand [25].

Additionally, if f takes 10^27^ m^−3^ [20], the value of A can be obtained. The calculation results show that the low saturation temperature and fast cooling rate mean that the formation rate of supersaturation is fast, resulting in a large kinetic factor A.

We can see from Table 4 that within the selected range of ethanol mass fraction, the interfacial energy γ first increases and then decreases with the increase of ethanol mass fraction, which may be due to the increase in the solubility of PMBA with the increase of ethanol mass fraction, and more energy is required to break the metastable state. The interfacial energy  γ decreases with the continuous increase in the ethanol mass fraction, probably because the amount of dissolved solute in the solution is large enough to easily aggregate to the critical size.

The value of the nucleation parameter *A*/*f* in Table 5 supports the above hypothesis; with the increase in the ethanol mass fraction, the attachment frequency of PMBA increases, and when the ethanol mass fraction continues to increase, the amount of dissolved solute increases, and it is easier to attach to the crystal surface, and the attachment efficiency increases, the corresponding attachment frequency decreases. Similar results can be observed in the nucleation behavior of methanol and water mixed solvents of glycine [27]. Under the same conditions, adding an anti-solvent to the solution will increase the supersaturation of the system. When the system is in the region of high supersaturation, homogeneous nucleation usually occurs; in contrast, heterogeneous nucleation is more likely to occur. For all binary solutions, the interfacial energy of the homogeneous process is greater than that of the heterogeneous process [28], which means that the homogeneous nucleation of PMBA in the solution with a 0.8 ethanol mass fraction is more likely than that in the other two solvent compositions.

**Figure 8:**
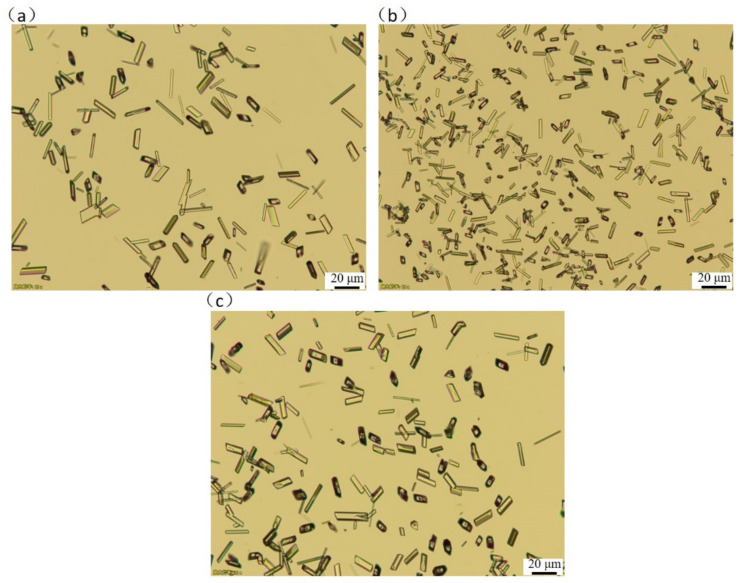
Crystal morphology of PMBA at ethanol mass fraction of (**a**) 0.6, (**b**) 0.8, and (**c**) 1.0 (saturation temperature 293.15 K, cooling rate 20 K/h, the scale in all figures is 20 µm).

The properties of the resulting crystals during crystallization are directly related to MSZW, and a narrower MSZW usually leads to an increase in particle size. A narrower MSZW means lower supersaturation during nucleation. The remaining supersaturation is consumed during the growth process, resulting in a larger particle size. To examine the effect of solution composition on MSZW, the crystals obtained by cooling crystallization of PMBA in different ethanol mass fractions were characterized by polarized light microscopy and are illustrated in Figure 8. Although the mass fraction of ethanol is different, the crystal habits of PMBA are similar. The MSZW was largest at a mass fraction of ethanol of 0.8, resulting in the smallest crystal size.

**Figure 9:**
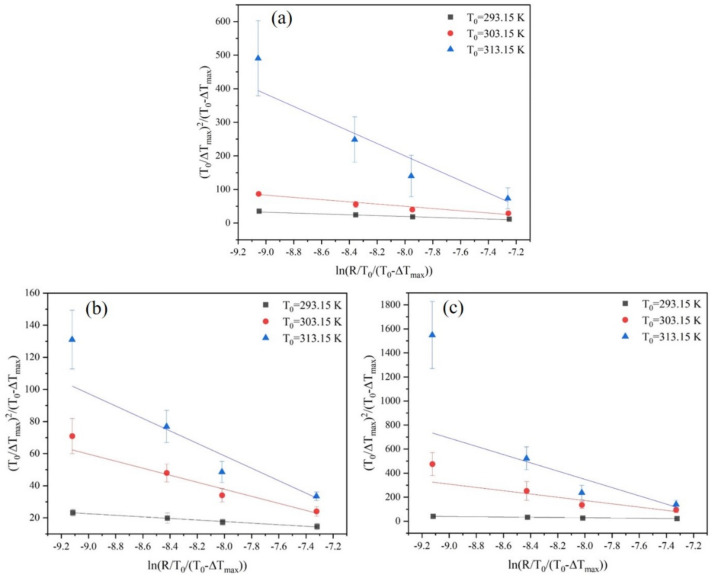
Relationship between MSZW and cooling rate at different $T_{0}$ fitted by the modified Sangwal model: (**a**) ethanol mass fraction of 0.6, (**b**) ethanol mass fraction of 0.8, and (**c**) ethanol mass fraction of 1.0.

In the Sangwal metastable zone model, the interfacial energy γ is related to $T_{0}$ and $T_{1}$, while the parameters *M* and *N* are independent of $T_{0}$ and $T_{1}$ can be obtained using the modified Sangwal metastable zone model. The MSZW data were fitted by the modified Sangwal model, as illustrated in Figure 9. Comparing the data in Table 4 and Table 5 with Table 6, it is found that the calculation of the interfacial energy γ and the nucleation parameter *A*/*f* are relatively similar, indicating that the modified Sangwal model not only simplifies the calculation process but can also fit the MSZW data well. Therefore, the data of interfacial energy γ and nucleation parameter *A*/*f* obtained by the modified Sangwal model are adopted in the subsequent discussion of critical parameters and nucleation kinetics.

### 4.5. Critical Nucleation Parameters and Nucleation Kinetics

To further elucidate the effect of $T_{0}$ and R on the nucleation behavior of PMBA in the ethanol-water system, the $r_{crit}$, $\Delta G_{crit}$ and J were calculated according to Equations (9)–(11) with the interfacial energy obtained by the modified Sangwal model.

The driving force Δμ [29] of a real solution can be expressed by the difference between liquid $\mu_{l}$ and solid $\mu_{s}$ on the basis of the theory of a regular solution.

(12) $$\Delta \mu =\mu_{l}-\mu_{s}=k_{B}T_{1}\ln S=k_{B}\frac{\Delta H_{s}}{R_{g}}\frac{\Delta T_{max}}{T_{0}}$$

**Figure 10:**
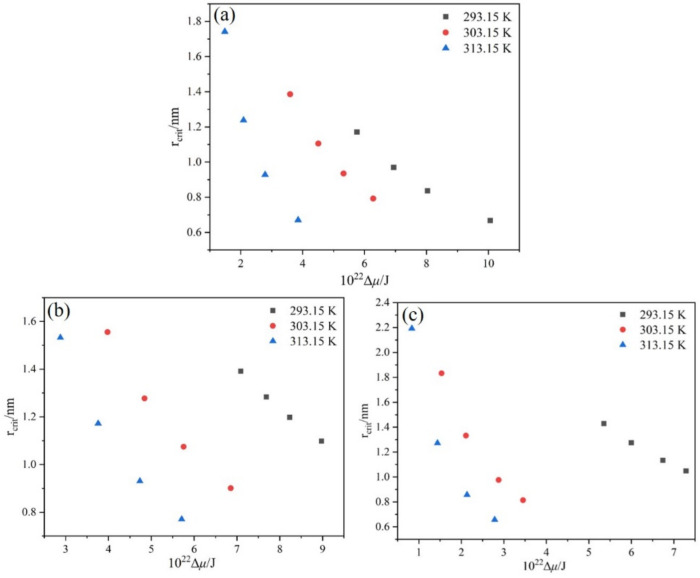
Relation between $r_{crit}$ and Δμ at different $T_{0}$: (**a**) ethanol mass fraction of 0.6, (**b**) ethanol mass fraction of 0.8, (**c**) ethanol mass fraction of 1.0.

**Figure 11:**
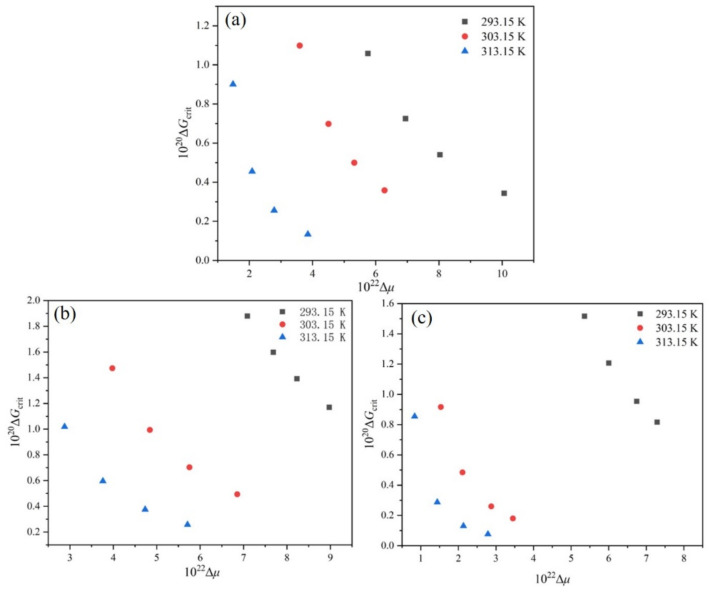
Relationship between $\Delta G_{crit}$ and Δμ at different $T_{0}$: (**a**) ethanol mass fraction of 0.6, (**b**) ethanol mass fraction of 0.8, (**c**) ethanol mass fraction of 1.0.

**Figure 12:**
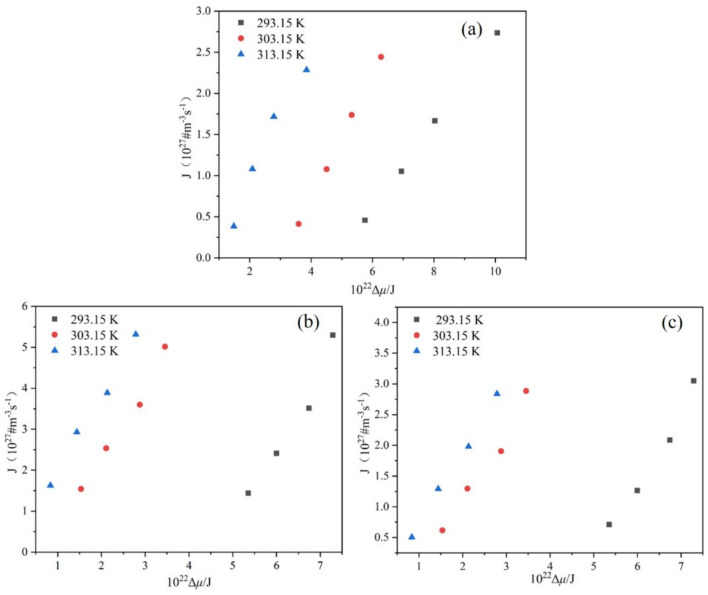
Relationship between J and Δμ at different $T_{0}$: (**a**) ethanol mass fraction of 0.6, (**b**) ethanol mass fraction of 0.8, (**c**) ethanol mass fraction of 1.0.

The dimensionless supersaturation *S* was calculated on the basis of the van’t Hoff equation, and the driving force Δμ was obtained using Equation (12). The $r_{crit}$, $\Delta G_{crit}$ and J are related to the Δμ, and Figure 10, Figure 11 and Figure 12 sketches these relationships.

Based on the information contained in Figure 10 and Figure 11, it is obvious that the critical nucleation parameters $r_{crit}$ and $\Delta G_{crit}$ both decrease with the Δμ increase at a given $T_{0}$, which implies that nucleation is easy to occur. Additionally, with the increase of $T_{0}$, the $r_{crit}$ and $\Delta G_{crit}$ required for nucleation decrease when the nucleation driving force is the same. Therefore, increasing the saturation temperature is favorable for nucleation. Meanwhile, with the same driving force Δμ, it is evident that the critical nucleation parameters $r_{crit}$ and $\Delta G_{crit}$ have the largest values at the ethanol mass fraction of 0.8. Therefore, it is not difficult to understand that the PMBA needs to overcome a higher nucleation barrier in the mixture solvent system with an ethanol mass fraction of 0.8. The result can be verified by interfacial energy data, and a higher value of γ usually leads to a wider MSZW [30], leading to smaller product sizes. In addition, we can also see from Figure 12 that when the driving force is constant, PMBA has the maximum nucleation rate in the solution with an ethanol mass fraction of 0.8. Interestingly, although the rate of nucleation increases monotonically with the nucleation driving force at the same $T_{0}$, the nucleation rate can remain almost unchanged when the nucleation driving force Δμ increases, and the saturation temperature $T_{0}$ decreases simultaneously. Thus, the nucleation rate lies in the interplay between Δμ and $T_{0}$.

**Figure 13:**
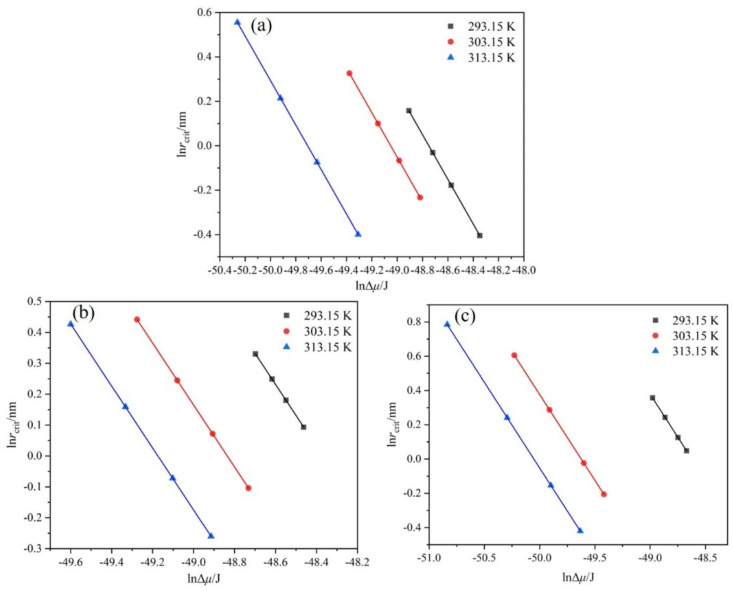
Relationship between $\ln r_{crit}$ and lnΔμ at different $T_{0}$ : (**a**) ethanol mass fraction of 0.6, (**b**) ethanol mass fraction of 0.8, (**c**) ethanol mass fraction of 1.0.

**Figure 14:**
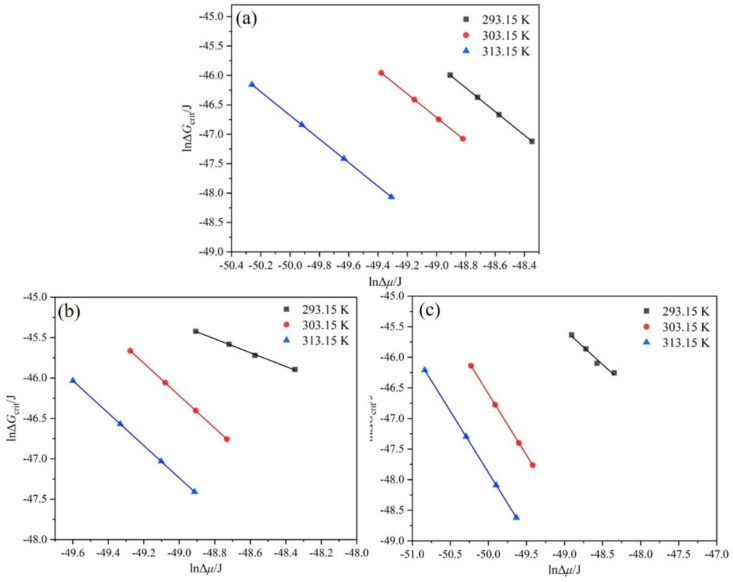
Relationship between $\ln\Delta G_{crit}$ and lnΔμ at different $T_{0}$ : (**a**) ethanol mass fraction of 0.6, (**b**) ethanol mass fraction of 0.8, (**c**) ethanol mass fraction of 1.0.

From Equations (9) and (10), we find that the critical nucleation parameters $r_{crit}$ and $\Delta G_{crit}$ are linearly correlated with $\frac{\gamma }{\Delta \mu }$ and $\frac{\gamma^{3}}{\Delta \mu^{2}}$, respectively. Therefore, we associate lnΔμ with $\ln r_{crit}$ and $\ln\Delta G_{crit}$ respectively and show them in Figure 13 and Figure 14. The results show that lnΔμ are negatively linearly correlated with both $\ln r_{crit}$ and $\ln\Delta G_{crit}$ at the same saturation temperature, which indicates that the interfacial energy does not rely on the nucleation driving force when the saturation temperature is the same. When the saturation temperature is different, the above relationship is broken, indicating that the interfacial energy is correlated with the nucleation driving force. These findings do not change with the change in the ethanol mass fraction. Therefore, it is vital to further study the relationship between interfacial energy and the nucleation driving force.

**Figure 15:**
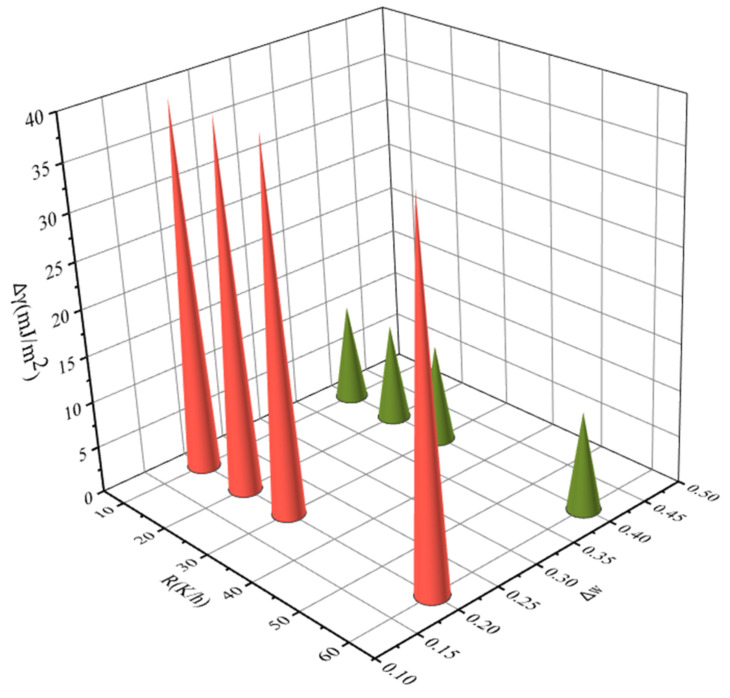
The relationship between interfacial energy γ and the change in ethanol mass fraction (saturated temperature 293.15 K, cooling rate 10, 20, 30, and 60 K/h, respectively).

**Figure 16:**
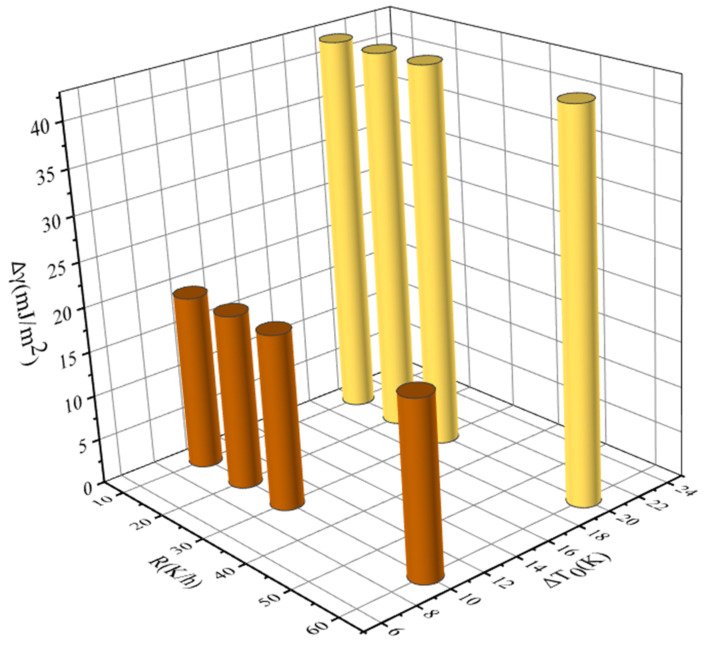
The relationship between interfacial energy and the change in saturated temperature (ethanol mass fraction 0.8, cooling rate 10, 20, 30, and 60 K/h, respectively).

**Figure 17:**
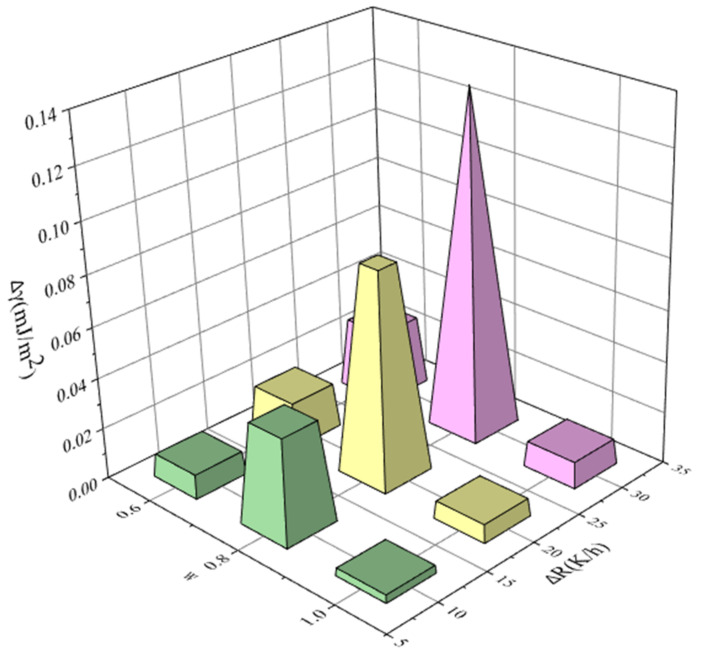
The relationship between interfacial energy and the change in cooling rate (saturated temperature 303.15 K, ethanol mass fraction 0.6, 0.8, and 1.0, respectively).

In order to further elucidate the effect of ethanol mass fraction, saturation temperature, and cooling rate on the MSZW of PMBA, we correlated the change of interfacial energy with the change of ethanol mass fraction, saturation temperature, and cooling rate, respectively. For example, there are three ethanol mass fractions: $\omega_{1}$ = 0.6, $\omega_{2}$ = 0.8 and $\omega_{3}$ = 1.0. We chose $\omega_{1}$ = 0.6 as a reference. Δω are 0.2 and 0.4, respectively. The results are sketched in Figure 15, Figure 16 and Figure 17 and show that the change of interfacial energy with saturation temperature is the most obvious, followed by the change of ethanol mass fraction, and the change of cooling rate has the least influence on the interfacial energy. Therefore, for the PMBA system, we proposed a preferential strategy to adjust MSZW by changing parameters; that is, changing the saturation temperature should be considered first, followed by changing the ethanol mass fraction, and finally changing the cooling rate.

## 5. Conclusions

In this paper, the MSZW of PMBA in an ethanol-water system was determined using the polythermal method, and different metastable zone models were used to analyze the nucleation behavior. The nucleation order *m* obtained by the Nývlt’s metastable zone model through fitting the MSZW data can roughly judge the nucleation mechanism. The nucleation of PMBA follows a progressive nucleation mechanism at a low saturation temperature and an instantaneous nucleation mechanism at a high saturation temperature. The data of γ and A/f were collected based on the Sangwal metastable zone model. The effects of cooling rate, saturation temperature, and different ethanol mass fractions on MSZW were also discussed. We found that γ decreases significantly with the increase of $T_{0}$, and increases slowly with the increase in cooling rate. While in the selected solvent composition range, the MSZW and interfacial energy reached the maximum at the mass fraction of ethanol 0.8. As a result, the nucleation of PMBA becomes difficult, resulting in the largest MSZW and the smallest crystal product size. The above results may be caused by the difficulty of breaking the metastable state due to solubility. The critical nucleation radius $r_{crit}$ and Gibbs free energy $\Delta G_{crit}$ calculated according to the Modified Sangwal model have the maximum values when the ethanol mass fraction is 0.8, indicating the PMBA needs to overcome a higher nucleation barrier. In addition, at a given driving force, PMBA had the maximum nucleation rate in the solution with an ethanol mass fraction of 0.8. Meanwhile, the nucleation rate lies in the interplay between Δμ and $T_{0}$. Furthermore, we noticed the lnΔμ are both very negatively significantly correlated with $\ln r_{crit}$ and $\ln\Delta G_{crit}$ at the same saturation temperature, which indicate that the interfacial energy γ is not dominated by the nucleation driving force when the saturation temperature is same. Finally, we proposed a preferential strategy for adjusting MSZW to guide the nucleation process of PMBA.

## Figures and Tables

**Table 1:** The dissolution enthalpy and dissolution entropy of PMBA under different ethanol masses.

ω	∆*H*_S_ (J/mol)	∆*S* (J/mol/K)	R^2^
0.6	35032.33	70.75	0.9919
0.8	30061.06	60.87	0.9966
1.0	29637.00	62.92	0.9974

**Table 2:** Metastable zone of PMBA in different ethanol mass fractions.

ω	$T_{0}/K$	$\Delta T_{max}/K$			
		R = 10 K/h	R = 20 K/h	R = 30 K/h	R = 60 K/h
0.6	293.15	2.900	3.350	3.650	4.075
0.6	303.15	1.875	2.350	2.776	3.275
0.6	313.15	0.800	1.125	1.500	2.075
0.8	293.15	3.575	3.875	4.150	4.525
0.8	303.15	2.075	2.525	3.000	3.575
0.8	313.15	1.550	2.025	2.550	3.075
1	293.15	2.700	3.025	3.400	3.675
1	303.15	0.800	1.100	1.500	1.800
1	313.15	0.450	0.775	1.150	1.500

**Table 3:** Nucleation order *m* fitted by Nývlt’s model.

ω	Nucleation Order *m*		
	*T*_0_ = 293.15 K	*T*_0_ = 303.15 K	*T*_0_ = 313.15 K
0.6	3.18	3.16	1.85
0.8	7.50	3.23	2.55
1.0	5.63	2.14	1.46

**Table 4:** The interfacial energies γ (mJ/m^2^) fitted by the Sangwal model.

ω	*T*_0_/K	γ (mJ/m^2^)			
		R = 10 K/h	R = 20 K/h	R = 30 K/h	R = 60 K/h
0.6	293.15	1.8450	1.8437	1.8426	1.8404
0.6	303.15	1.3658	1.3650	1.3644	1.3636
0.6	313.15	0.7090	0.7088	0.7085	0.7080
0.8	293.15	2.3195	2.3187	2.3179	2.3169
0.8	303.15	1.4552	1.4545	1.4537	1.4528
0.8	313.15	1.0368	1.0363	1.0357	1.0351
1.0	293.15	1.774	1.7733	1.7726	1.7720
1.0	303.15	0.6518	0.6516	0.6513	0.6511
1.0	313.15	0.4246	0.4244	0.4243	0.4241

**Table 5:** Nucleation parameter *A*/*f* fitted by the Sangwal model.

ω	*T*_0_/K	*A*/*f* (s^−1^)			
		R = 10 K/h	R = 20 K/h	R = 30 K/h	R = 60 K/h
0.6	293.15	6.4345	6.4478	6.4601	6.4830
0.6	303.15	5.7808	5.7899	5.7981	5.8078
0.6	313.15	3.1034	3.1066	3.1104	3.1161
0.8	293.15	45.8461	45.8936	45.9373	45.9970
0.8	303.15	26.4174	26.4570	26.4989	26.5497
0.8	313.15	3.5892	3.5947	3.6008	3.6069
1.0	293.15	16.9758	16.9949	17.0169	17.0330
1.0	303.15	2.9710	2.9740	2.9779	2.9809
1.0	313.15	2.0819	2.0841	2.0866	2.0890

**Table 6:** *M*, *N*, γ and *A*/*f* fitted by the modified Sangwal model.

Ethanol Mass Fraction	$T_{0}/K$					
	293.15	303.15	313.15	293.15	303.15	313.15
	*N*			*M*		
0.6	−13.22	−32.68	−234.00	−85.41	−213.90	−1671.07
0.8	−4.90	−20.13	−55.03	−21.57	−99.58	−380.02
1.0	−10.66	−215.71	−780.63	−57.51	−1535.38	−5809.89
	γ (mJ/m^2^)			*A*/*f*		
0.6	1.8498	1.3681	0.7098	6.5891	6.0545	3.3360
0.8	2.3254	1.4519	1.0384	44.3009	25.6925	3.6232
1.0	1.7777	0.6524	0.4249	16.1821	2.8894	2.0881

## Data Availability

The data presented in this study are available on request from the State Key Laboratory of Chemical Engineering, School of Chemical Engineering and Technology, Tianjin University.

## Sample Availability

Not available.

## References

[1] Derdoura L., Skliarb D. (2014). A review of the effect of multiple conformers on crystallization from solution and strategies for crystallizing slow inter-converting conformers. Chem. Eng. Sci..

[2] Nangia A.K., Desiraju G.R. (2019). Crystal Engineering: An Outlook for the Future. Angew. Chem. Int. Ed..

[3] Collins K.D. (2004). Ions from the Hofmeister series and osmolytes: Effects on proteins in solution and in the crystallization process. Methods.

[4] Kashchiev D., Rosmalen G.M. (2003). Review: Nucleation in solutions revisited. Cryst. Res. Technol..

[5] Mitchell N.A., Frawley P.J. (2010). Nucleation kinetics of paracetamol-ethanol solutions from metastable zone widths. J. Cryst. Growth.

[6] Lenka M., Sarkar D. (2014). Determination of metastable zone width, induction period and primary nucleation kinetics for cooling crystallization of l-asparaginenohydrate. J. Cryst. Growth.

[7] Shiau L.D., Lu T.S. (2014). A model for determination of the interfacial energy from the induction time or metastable zone width data based on turbidity measurements. CrysEngComm.

[8] Liang K.P., White G., Wilkinson D., Ford L.J., Roberts K.J., Wood W.M.L. (2004). An examination into the effect of stirrer material and agitation rate on the nucleation of l-glutamic acid batch crystallized from supersaturated aqueous solutions. Cryst. Growth Des..

[9] Peters B. (2011). Supersaturation rates and schedules: Nucleation kinetics from isothermal metastable zone widths. J. Cryst. Growth.

[10] Yuan Y., Leng Y., Huang C., Yue M., Tan Q. (2015). Effects of cooling rate, saturation temperature, and agitation on the metastable zone width of dl-malic acid-water system. Russ. J. Phys. Chem. A.

[11] Rajesh N.P., Perumal C.K.L., Raghavan P.S., Ramasamy P. (2001). Effect of urea on metastable zone width, induction time and nucleation parameters of ammonium dihydrogen orthophosphate. Cryst. Res. Technol..

[12] Yang J., Xu S., Wang J., Gong J. (2020). Nucleation behavior of ethyl vanillin: Balance between chemical potential difference and saturation temperature. J. Mol. Liq..

[13] Xu S., Bu Y., Jiang S., Yang P., Wang Y. (2021). Insights into the Role of Solvents in Nucleation Kinetics of Glutaric Acid from Metastable Zone Widths. Ind. Eng. Chem. Res..

[14] Wang Y., Chuai X., Li Y., Guo J., Yang J., Liu Z., Xu S. (2022). Nucleation Behaviors of Adipic Acid in Different Polarity Solvent Based on Metastable Zone Width. Crystals.

[15] Zhao Y., Hou G., Kamaraju V.K., He Y., Power G., Glennon B. (2020). Primary Nucleation of Benzoic Acid in Aqueous Ethanol Solution. Ind. Eng. Chem. Res..

[16] Gandhi P.J., Murthy. Z.V.P. (2013). Transmission of *p*-anisic acid through nanofiltration and goat membranes. Desalination.

[17] Nývlt J. (1968). Kinetics of nucleation in solutions. J. Cryst. Growth.

[18] Kashchiev D., Borissova A., Hammond R.B., Robertsb K.J. (2010). Effect of cooling rate on the critical undercooling for crystallization. J. Cryst. Growth.

[19] Sangwal K. (2009). A novel self-consistent Nývlt-like equation for metastable zone width determined by the polythermal method. Cryst. Res. Technol..

[20] Sangwal K. (2009). Novel approach to analyze metastable zone width determined by the polythermal method: Physical interpretation of various parameters. Cryst. Growth Des..

[21] Sangwal K. (2011). Some features of metastable zone width of various systems determined by polythermal method. CrysEngComm.

[22] Mullin J.W., Mullin J.W. (2001). Nucleation. Crystallization.

[23] Xu S., Wang J., Zhang K., Wu S., Liu S., Li K., Yu B., Gong J. (2016). Nucleation behavior of eszopiclone-butyl acetate solutions from metastable zone widths. Chem. Eng. Sci..

[24] Xiong L., Zhou L., Zhang X., Zhang M., Hou B., Bao Y., Du W., Su W., Zhang S., Yin Q. (2018). Determination of metastable zone widths and nucleation behavior of aspirin in acetic acid and acetic anhydride binary solvent mixture. J. Mol. Liq..

[25] Peng H., Tian N., Yu C., Gao Y., Li K., Yan H., Zhao P., Wu S., Chen M., Gong J. (2022). Insights into the Role of Dipentaerythritol in the Thermodynamics and Nucleation Behavior of a Pentaerythritol-Water System. Cryst. Growth Des..

[26] Sangwal K. (2011). Recent developments in understanding of the metastable zone width of different solute-solvent systems. J. Cryst. Growth.

[27] Ramakers L.A.I., McGinty J., Beckmann W., Beckmann W., Levilain G., Lee M., Wheatcroft H., Houson I., Sefcik J. (2020). Investigation of Metastable Zones and Induction Times in Glycine Crystallisation across Three Different Antisolvents. Cryst. Growth Des..

[28] Zou F., Zhuang W., Chen Q., Yang P., Lin C., Jiao P., Zhou J., Wu J., Ying H. (2016). Solvent effects on nucleation of disodium guanosine 5′-monophosphate in anti-solvent/water mixtures. CrystEngComm.

[29] Granberg R.A., Ducreux C., Gracin S., Rasmuson A.C. (2001). Primary nucleation of paracetamol in acetone-water mixtures. Chem. Eng. Sci..

[30] Sullivan R.A., Davey R.J., Sadiq G., Dent G., Back K.R., ter Horst J.H., Toroz D., Hammond R.B. (2014). Revealing the Roles of Desolvation and Molecular Self-Assembly in Crystal Nucleation from Solution: Benzoic and *p*-Aminobenzoic Acids. Cryst. Growth Des..

